# Gibberellic Acid Production by Different Fermentation Systems Using Citric Pulp as Substrate/Support

**DOI:** 10.1155/2017/5191046

**Published:** 2017-09-07

**Authors:** Juliana de Oliveira, Cristine Rodrigues, Luciana P. S. Vandenberghe, Marcela C. Câmara, Nelson Libardi, Carlos R. Soccol

**Affiliations:** Bioprocess Engineering and Biotechnology Department, Federal University of Paraná (UFPR), Curitiba, PR, Brazil

## Abstract

Gibberellic acid (GA_3_) is an important phytohormone, a member of gibberellins family, which acts as a promoter and regulator of plant growth. This study aimed to evaluate GA_3_ production by* Fusarium moniliforme* LPB03 and* Gibberella fujikuroi* LPB06 using different techniques of fermentation, solid state fermentation (SSF), submerged fermentation (SmF), and semisolid state fermentation (SSSF), and different types of bioreactors. In all techniques, citric pulp (CP), a subproduct obtained from the extraction of orange juice, was employed as the substrate/support. GA_3_ production by SSF reached 7.60 g kg^−1^ and 7.34 g kg^−1^ in Erlenmeyer flasks and column bioreactors, respectively. For SmF, the highest concentration of GA_3_ obtained was 236.00 mg L^−1^ in Erlenmeyer flasks, 273.00 mg L^−1^ in a 10 L stirred tank reactor (STR), and 203.00 mg L^−1^ in a 1.5 L bubble column reactor (BCR). SSSF was conducted with a CP suspension. In this case, GA_3_ concentration reached 331.00 mg L^−1^ in Erlenmeyer flasks and 208 mg L^−1^ in a BCR. The choice of the fermentation technique is undoubtedly linked to the characteristics and productivity of each process. The methods studied are inexpensive and were found to produce good proportions of GA_3_, making them suitable for several applications.

## 1. Introduction

Gibberellins (GAs) consist of a family of diterpenoid acids, an important group of phytohormones that exercise different effects on growth and development of plants, such as germination, cell elongation, expansion of leaves, and development of flowers [1–3]. Similar to auxins, they stimulate the activity of transference, generating higher development of xylem and phloem in ligneous plants [4–6]. These properties make gibberellins a valuable tool in agriculture to increase crop yields [7, 8].

Among the 136 GAs isolated, gibberellic acid (GA_3_) has received the most attention. The use of GA_3_ has been extensively studied in different crop plants, and the results vary depending on the plant species, form of application, and concentration of this hormone [9–14].

GAs are found in plants, algae, fungi, and bacteria. However, due to high concentrations in fungus, industrial production of GAs is performed by submerged fermentation of the ascomycetous fungus* G. fujikuroi.* Production by plant extraction is not viable because of low concentrations of GAs, which contributes to waste generation [15]. While the chemistry, biosynthesis, mode of action, and relationships between structure and activity of GAs have already been extensively investigated [7, 16, 17], little is known about the production of GAs by fermentation [18].

There are several studies evaluating the decrease in production costs of GA_3_ with the use of different techniques, such as screening and genetic manipulation of microorganisms, optimization of culture conditions and nutrients, development of new fermentative processes, minimization of extraction costs, and use of cheaper substrates such as agroindustrial byproducts and wastes [19]. These supports include straws, husks, bagasses, and brans, which have high fiber content and allow the possibility of working with high humidity content [20, 21]. Some wastes have already been used for GA_3_ production, such as wheat bran [22] and rice flour [8, 23].

Citric pulp (CP) is a subproduct obtained through the treatment of liquid and solid wastes remaining from the extraction of orange juice. These wastes include peel, seed, and orange pulp, which constitute 50% of the fruit weight [25]. The estimate of global orange juice production (at 65 degrees' brix) for 2016/17 is two million metric tons [26]. For this purpose, it is necessary to use around 22 million metric tons of oranges; thus, 11 million tons of citric pulp is generated. In Brazil alone, the estimate is 12.9 million tons of oranges for processing [26] and around 6.5 million tons of citric pulp generated. These citric wastes are rich in carbohydrates and other nutrients and are a viable substrate for solid state fermentation (SSF) and other fermentation techniques as submerged fermentation (SmF) and semisolid state fermentation (SSSF), after a physical and/or chemical pretreatment.

This study aimed to evaluate the production of GA_3_ using CP as the substrate/support for SSF, SmF, and SSSF, using different types of bioreactors.

## 2. Materials and Methods

### 2.1. Strain Maintenance

The strains* Fusarium moniliforme* LPB03 and* Gibberella fujikuroi* LPB06 were conserved in assay tubes previously prepared in potato dextrose agar (PDA) slants and incubated for six days at 28–30°C. Strains were then maintained at 4°C, for up to three months, and periodically renovated.

### 2.2. Substrates

The composition of CP is shown in Table 1. The pH of CP (5.76) is close to that normally used for the GA_3_ production process. The amount of sugar supports the use of CP as a substrate for fermentation. In addition, the carbon : nitrogen (C : N) ratio is high, which favors the production of GA_3_ [23]. In relation to ions, phosphate and sulfate are the most prevalent in the media composition for producing GA_3_ [8, 18], and nitrates are also used in synthetic media.

CP was initially dried, ground in a records mill, and classified in order to obtain particle sizes less than 5 mm. Solid CP was used in SSF.

For SmF, an aqueous extract of CP (AECP) was prepared using the ratio 1 : 10 of dry CP and water (w/v). The suspension was heated in a boiling water bath for 30 min and then filtrated in order to remove the suspended solids, thus obtaining the AECP.

For SSSF, an aqueous suspension of grinded CP (particle size 2–2.8 mm) was prepared, containing 5% of suspended solids.

### 2.3. Inoculum Preparation

Inoculum of* G. fujikuroi* LPB06 was grown in 250 mL Erlenmeyer flasks containing 100 mL of 5% (w/v) AECP, at 28°C, in a rotary shaker at 120 rpm, for four days.

For* F. moniliforme* LPB 03, 10% (w/v) AECP was supplemented with sucrose (30 g L^−1^). The strain was then transferred and grown in a rotary shaker at 120 rpm for four days, at 28°C [27].

### 2.4. Solid State Fermentation (SSF)

#### 2.4.1. SSF in Erlenmeyer Flasks (Aeration by Diffusion)

CP was impregnated with a nutritive solution containing 1.5 g L^−1^ urea and 1.5 g L^−1^ MgSO_4_·7H_2_O, in order to produce initial moisture of 75%. Initial pH was 5.5–5.8, which corresponds to the natural pH of CP. The support was inoculated with 10% (v/w) of the mycelia suspension. After homogenization, the inoculated support was transferred to Erlenmeyer flasks (250 mL). These conditions were previously optimized (data not shown). A kinetic study was performed over seven days, at 29°C, in triplicate. Analysis of GA_3_ concentration was performed every 24 h.

#### 2.4.2. SSF in Column Bioreactors (Forced Aeration)

**Figure 1:**
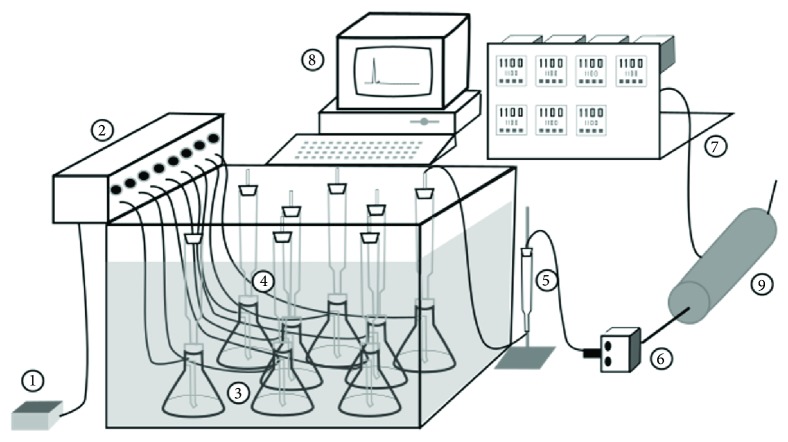
Data acquisition system to determine O_2_ consumption and CO_2_ production during SSF in column bioreactor: ① air pump; ② air distribution system; ③ humidifiers; ④ fermentation columns immersed in a water bath with controlled temperature reactor; ⑤ filter; ⑥ structure with the sensors; ⑦ controllers panel; ⑧ computer with data acquisition and control software; ⑨ cylindrical sensors base, where the sensors are installed: CO_2_ and O_2_, humidity, and outlet temperature. Source: [24].

SSF was conducted in 0.25 L column bioreactors (4 cm diameter and 20 cm length), containing 30 g of dry CP, to study the influence of forced aeration on GA_3_ production. The initial moisture of CP was adjusted to 70% (v/w) with a nutritive solution composed of 1.5 g L^−1^ urea and 1.5 g L^−1^ MgSO_4_·7H_2_O. The inoculated substrate was transferred to column bioreactors, which were then placed into a water bath with temperature control at 29°C. Saturated air was pumped continually through the columns in order to control substrate temperature and moisture. The airflow was controlled at 30 mL min^−1^. The microorganism respiratory metabolism was evaluated by determining the O_2_ consumption and CO_2_ production (Figure 1) [24]. Fermentation was carried out for seven days. Analysis was performed every 24 h.

### 2.5. GA_3_ Production in Submerged Fermentation (SmF)

#### 2.5.1. SmF in Erlenmeyer Flasks

GA_3_ production by SmF was carried out in 250 mL Erlenmeyer flasks containing 50 mL of medium composed of 10% (w/v) AECP and 0.5 g L^−1^ of MgSO_4_·7H_2_O. The inoculum rate was 10% (v/v) of mycelia suspension, previously grown in medium composed of 5% (w/v) AECP. The study was conducted at 29°C and 120 rpm in a rotary shaker, over 240 h. Samples were withdrawn every 24 h for analysis of GA_3_ concentration.

#### 2.5.2. SmF in Stirred Tank Reactor (STR)

Batch fermentation was conducted in a 10 L STR (New Brunswick Scientific, Bioflo 110) with 6 L working volume medium composed of 10% (w/v) AECP (18 g L^−1^ of total sugars) and 0.5 g L^−1^ of MgSO_4_·7H_2_O. The medium was inoculated with mycelia suspension of* G. fujikuroi* at a rate of 10% (v/v). GA_3_ production was performed at 29°C, with an initial pH of 5.0, agitation of 500 rpm, and an aeration rate of 1 L min^−1^. Fermentation was carried out over 240 h, and samples were withdrawn every 24 h for analysis.

#### 2.5.3. SmF in Bubble Column Reactor (BCR)

**Figure 2:**
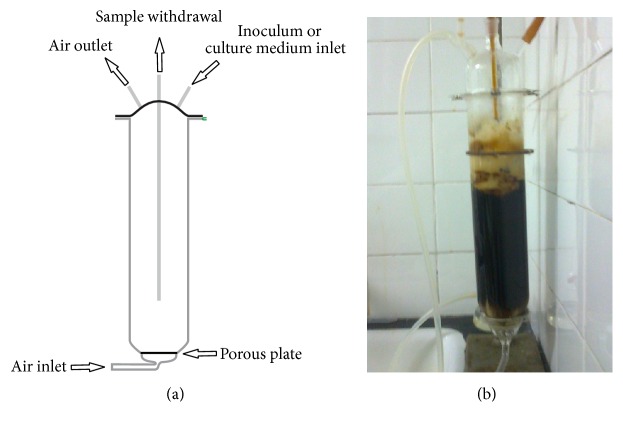
Design of 1.5 L bubble column reactor (BCR) for SFm and SSSF. (a) Design of reactor: borosilicate glass with a total volume of 1.5 L and working volume of 1 L (diameter of 80 mm and a height of 300 mm). (b) Reactor during fermentation for GA_3_ production by SmF.

GA_3_ production was scaled up in a bubble column reactor (BCR), under pneumatic agitation. The BCR is a cylindrical tube of borosilicate glass with a total volume of 1.5 L and working volume of 1 L (diameter of 80 mm and a height of 300 mm) (Figure 2). Sterile air is injected through the inlet from the bottom of the column, passing through a porous plate and forming bubbles. The top has three connections: one lateral connection for air outlet, one lateral connection for inoculum or culture medium feeding, and a central connection for sampling during fermentation.

SmF was conducted for 216 h, with production medium composed of 10% (w/v) AECP and 0.5 g L^−1^ of MgSO_4_. The temperature was maintained at 29°C, with a 10% (v/v) inoculum rate, an initial pH adjusted to 5.0, and an aeration rate of 1 L min^−1^.

### 2.6. Production of GA_3_ by Semisolid State Fermentation (SSSF)

#### 2.6.1. SSSF in Erlenmeyer Flasks

A suspension with 5% (w/v) of CP solids was used for GA_3_ production. 100 mL of the medium, added to 20 g L^−1^ of sucrose and 0.6 g L^−1^ of urea, was distributed in 250 mL Erlenmeyer flasks. Flasks were inoculated with a mycelia suspension of* G. fujikuroi* at a rate of 10% (v/v). SSSF was performed at 29°C and 120 rpm in an orbital shaker. Samples were withdrawn every 24 h for GA_3_ analysis.

#### 2.6.2. SSSF in BCR

GA_3_ production was performed in a 1.5 L BCR containing 1 L of a culture medium (5% (w/v) CP suspension added to 20 g L^−1^ of sucrose and 0.6 g L^−1^ of urea). Assays were conducted at 29°C with a 10% (v/v) inoculum rate, an initial pH of 5.0, and an aeration rate of 1 L min^−1^, for 216 h.

### 2.7. Analytical Procedure

After SSF, GA_3_ was extracted with phosphate buffer (pH 8.0) and filtered through membranes. After SmF and SSSF, the broth was filtered to remove biomass and CP particles. GA_3_ was quantified by spectrophotometry at 254 nm, according to Holbrook et al. [28]. Biomass was determined by the ergosterol method [29].

The respiratory metabolism of the microorganism in SSF was evaluated by determining the O_2_ consumption and CO_2_ production, as an indirect method for biomass evaluation. The data acquisition system was composed of a Software, Fersol 2, and sensors [30]. These sensors acquired online data for the fermentation process parameters (O_2_, CO_2_, temperature, and humidity).

## 3. Results and Discussion

### 3.1. GA_3_ Production by SSF Using* F. moniliforme* LPB 03

**Figure 3:**
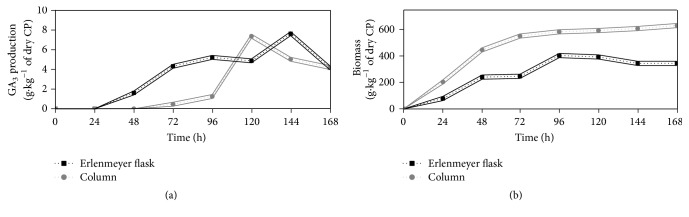
GA_3_ production by SSF in flask and column. (a) GA_3_ production profile. (b) Growth profile of* F. moniliforme* in CP during the GA_3_ production. Values and error bars represent the mean and standard deviation of triplicate experiments.

GA_3_ production was carried out by SSF with the strain* F. moniliforme LPB 03 *and with two different bioreactors, Erlenmeyer flasks (aeration by diffusion) and column bioreactors (forced aeration) (Figure 3). The highest productivity of GA_3_ (0.06 g kg^−1^ h^−1^ of dry CP), with a production of 7.34 g kg^−1^, was observed in column bioreactors after 120 h. In Erlenmeyer flasks, the production was 7.60 g kg^−1^ of dry CP in 144 h, which represents a productivity of 0.05 g kg^−1^ h^−1^. This means that the production with forced aeration led to a gain of 13.72% compared to the results obtained in Erlenmeyer flasks. This increase may have occurred due to differences in bioreactor configuration and the influence of aeration and light. Such a positive effect of aeration on GA_3_ production was demonstrated by Machado et al. [31], who achieved 0.49 g kg^−1^ of dry substrate in Erlenmeyer flasks and 0.93 g kg^−1^ of dry substrate in aerated columns, corresponding to an increase of 87%. According to Taiz and Zeiger [32], the production of gibberellins is also induced by light. Column bioreactors are more exposed to light; this may be one reason for the better GA_3_ synthesis. Forced aeration through a solid fixed bed is also responsible for heat dissipation, which is generated by the exothermic reaction of the fungus metabolism. When heat is not dissipated, high temperatures are attained, and these could inhibit growth. The configuration of the column bioreactor (forced aeration and direct exposure to sunlight) likely favored the production of biomass, which was higher than that observed in Erlenmeyer flasks (aeration by diffusion without illumination). The growth profile observed in column type bioreactors was significantly accelerated compared to Erlenmeyer flasks. The exponential growth phase with forced aeration occurred from 24 to 96 h, whereas with aeration by diffusion it was observed between 24 and 120 h.

**Figure 4:**
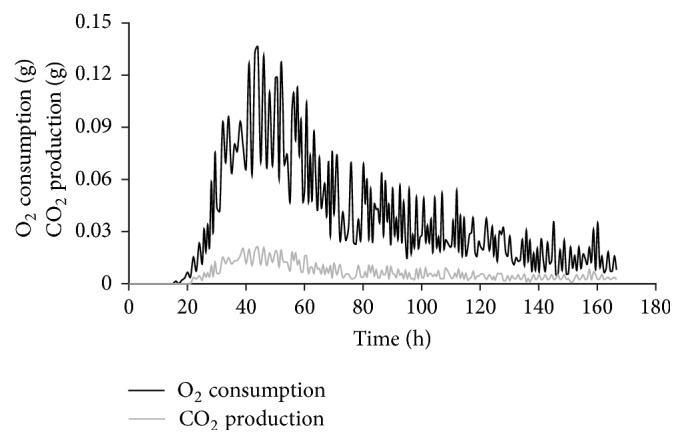
O_2_ consumption and CO_2_ production during SSF by* F. moniliforme* in column bioreactor.

The respiratory metabolism of the fungus is closely linked to growth of the microorganism [33] (Figure 4). After 24 h of fermentation, it was possible to see the beginning of a more pronounced cellular respiration, due to the fact that the organism was already adapted to the environment. The highest rates of respiration (O_2_ consumption and CO_2_ production) are thought to be related to the exponential growth phase, which was expected. After 48 h of fermentation, there was a drop in O_2_ consumption and CO_2_ production, which is probably linked to the beginning of the stationary phase. After 70 h, the respiration of the fungus remained almost constant until the end of fermentation for fungus maintenance and the synthesis of the secondary metabolite, or GA_3_.

### 3.2. GA_3_ Production by SmF Using* G. fujikuroi* LPB 06

**Figure 5:**
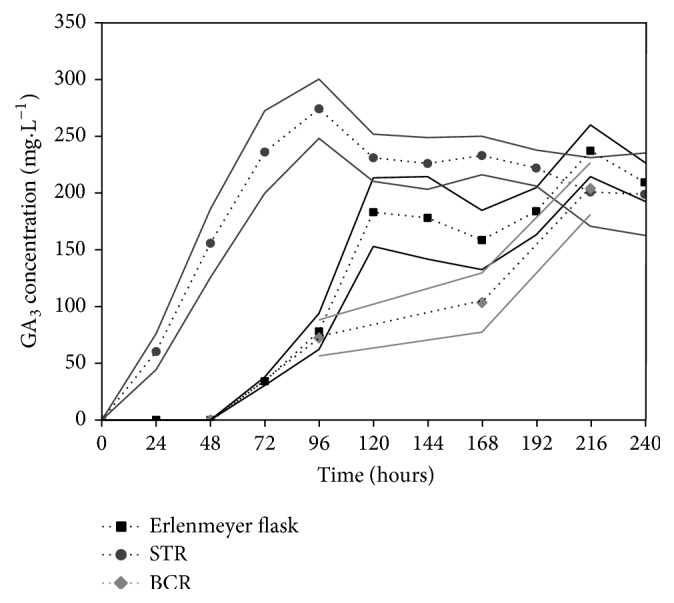
GA_3_ production by* G. fujikuroi* using SmF in Erlenmeyer flask, STR, and BCR. Values and error bars represent the mean and standard deviation of triplicate experiments.

GA_3_ production by* G. fujikuroi* LPB 06 was performed by SmF in Erlenmeyer flasks, with CPAE. The strain was previously screened and showed the best adaptation to this fermentation system (data not shown). The synthesis of GA_3_ started after 48 h (Figure 5), reaching the highest concentration (236.00 mg L^−1^ or 2.73 g kg^−1^ of dry CP) at 216 h, with a productivity of 1.09 mg L^−1^ h^−1^. However, the highest productivity, 1.52 mg L^−1^ h^−1^, was obtained at 120 h of fermentation. GA_3_ concentration did not increase continuously during fermentation, and this behavior was also observed during GA_3_ production by SSF.

The scale-up of GA_3_ production by SmF in a 6 L (working volume) STR (Figure 5) provided higher GA_3_ concentrations and productivity, 273.00 mg L^−1^ (3.17 g kg^−1^ of dry CP) and 2.8 mg L^−1^ h^−1^, respectively, after 96 h of fermentation. In BCR, using the same aeration rate that was used in STR, 1 L min^−1^, GA_3_ production started after 96 h of fermentation, and the highest GA_3_ concentration reached 203.00 mg L^−1^ (0.94 mg L^−1^ h^−1^) in 216 h.

GA_3_ production reached a higher concentration and productivity in STR than in Erlenmeyer flasks and BCR. The increase of GA_3_ production and productivity in STR is related to the influence of the forced aeration and mechanical agitation, which promotes better homogeneity and better mass and oxygen transfer during the fermentation. According to Escamilla et al. [23] and Lale and Gadre [34], GA_3_ production is influenced by the high concentration of dissolved oxygen in the medium. The influence of agitation and aeration rate on GA_3_ production by STR has been observed in previous work. Durán-Páramo et al. [35] used a 3.5 L (working volume) STR, with 200 rpm and 0.3 vvm (volume of air per volume of medium per minute), which reached 206.00 mg L^−1^ (0.60 mg L^−1^ h^−1^) of GA_3_ production with a medium composed of 25.00 g L^−1^ glucose as the carbon source. On the contrary, Shukla et al. [8] used a 1.80 L (working volume) STR and higher conditions of agitation speed and aeration (700 rpm and 1 vvm), which reached 1.00 g L^−1^ (5.90 mg L^−1^ h^−1^) of GA_3_, using a medium composed of 80.00 g L^−1^ of glucose as the carbon source. In the present study, 273.00 mg L^−1^ was obtained (2.80 mg L^−1^ h^−1^) using a culture medium composed of an agroindustrial subproduct (AECP) without the addition of a carbon source, which represents an advantage in terms of process costs.

Despite the aeration rate of 1 vvm, GA_3_ production was lower in BCR than in STR. This bioreactor did not provide very good homogeneity and mass transfer. This was probably due to the higher size of fungi pellets (data not shown), which led to mass transfer limitations and lowering of nutrients and oxygen levels, especially in the center region of pellets [36]. The influence of pellet size on GA_3_ production was described by Escamilla et al. [23]. Chavez-Parga et al. [37] used a 3.5 L air lift bioreactor for GA_3_ production, with an aeration rate of 1.6 vvm (5.6 L min^−1^). In this case, GA_3_ production reached 100.00 mg L^−1^ (0.30 mg L^−1^ h^−1^). In the airlift bioreactor, the agitation of broth is pneumatic, similar to BCR; however, the airlift bioreactor is composed of an internal tube, which organizes and directs the airflow in the system. GA_3_ concentration and productivity obtained in the present study were around two and three times higher than those obtained by Chavez-Parga et al., respectively.

### 3.3. GA_3_ Production by SSSF Using* G. fujikuroi* LPB 06

**Figure 6:**
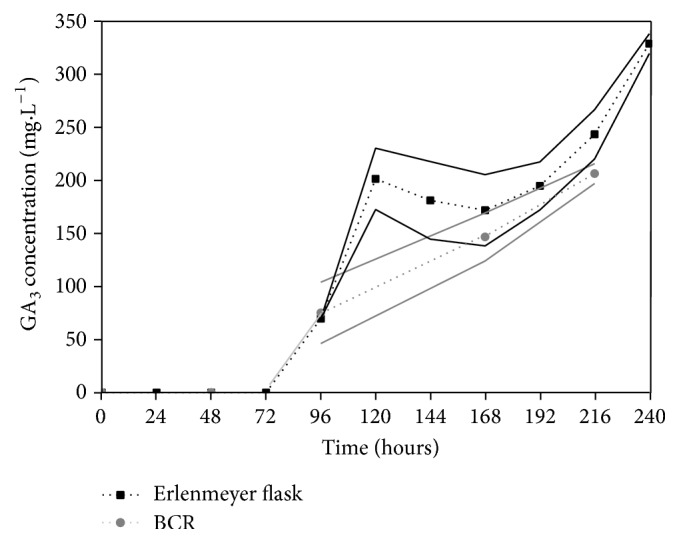
GA_3_ production by* G. fujikuroi *using SSSF in Erlenmeyer flask and BCR. Values and error bars represent the mean and standard deviation of triplicate experiments.

GA_3_ production was also carried out by SSSF in Erlenmeyer flasks using CP suspended solids (Figure 6), promoting a maximum GA_3_ concentration of 331.00 mg L^−1^ (7.70 g kg^−1^ dry CP) after 240 h of fermentation, or a productivity of 1.38 mg L^−1^ h^−1^. In terms of dry substrate, GA_3_ production by SSSF was similar to the production obtained by SSF.

**Figure 7:**
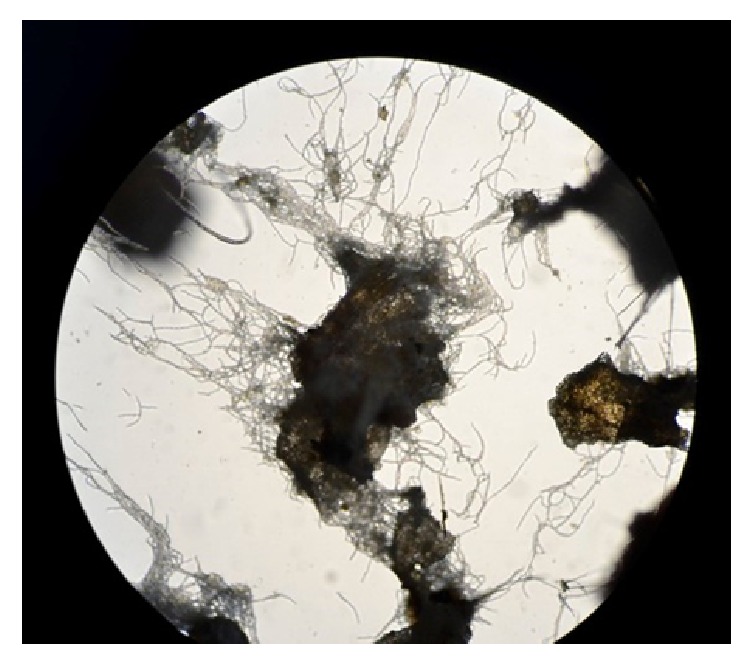
Microscopy (200x) of CP particle after fermentation to evaluate the adhesion of fungus to CP particle as a support.

The process was also performed in a BCR, where GA_3_ production reached 208.00 mg L^−1^ (productivity of 0.96 mg L^−1^ h^−1^), after 216 h of fermentation; this is equivalent to the results obtained in BCR by SmF. SSSF presented some advantages when compared to SmF because CP is directly in the medium. Therefore, the substrate does not need chemical or enzymatic pretreatment before fermentation. CP particles provide nutrients as well as support to filamentous fungus during fermentation. The adhesion of fungus to CP particles as a support is evidenced in Figure 7.

### 3.4. Comparison between Different Fermentation Techniques for GA_3_ Production

GA_3_ production was performed by SSF, SmF, and SSSF, using different bioreactors, and with CP as the substrate/support (Table 2). The highest GA_3_ production and productivity was obtained using SSF (7.60 g kg^−1^ of dry CP). In terms of GA_3_ production by kg of dry CP, SSSF presented comparable results to SSF (7.60 g kg^−1^ of dry CP).

The evaluation of the production of biocompounds by SSF and SmF has been studied, and certain bioactive compounds have been found to be produced in higher quantities in SSF, whereas other compounds have been extracted using SmF [38]. Higher production of biomolecules, such as enzymes [39, 40] and organic acids [41], is generally obtained through SSF fermentation systems. However, this fermentation technique and the SSF bioreactors are not easily scaled up. SSF currently promotes higher concentrations of biomolecules, in comparison to SmF or SSSF, due to the higher concentrations of substrate under limited free water conditions. Moreover, SSF reproduces a natural environment for filamentous fungi growth [42–44]. These fermentation systems present advantages, such as the use of solid agroindustrial wastes/subproducts as substrates in their natural form, which contributes to less wastewater production [43, 44]. In this way, CP proved to be a very good substrate for GA_3_ production in different bioreactors and with different fermentation techniques, which provides a very positive outlook for this process.

## 4. Conclusions

The results in this study demonstrate that SSF provides the highest GA_3_ production and productivity by* F. moniliforme*. Furthermore, this technique uses a low amount of water, which consequently lowers the cost and waste generation associated with this process. In the studied bioreactor systems, GA_3_, which is a high-value molecule, was produced in good proportions. Medium to high concentrations of the product can be achieved with simple processes employing CP as a substrate. CP is a subproduct of the orange juice industry, which is generated in abundance. Therefore, the costs of GA_3_ production could be reduced, making its application viable in different important agriculture cultivars.

## Figures and Tables

**Table 1:** Physical-chemical composition of citric pulp.

Component	Amount
Moisture	11.42%
Ash	7.89%
Protein	6.30%
Total sugar	20.22%
Fluoride	100.11 mg kg^−1^
Nitrate	375.76 mg kg^−1^
Phosphate	149.16 mg kg^−1^
Sulphate	201.74 mg kg^−1^
Fe	174.5 mg kg^−1^
Cu	8.4 mg kg^−1^
Zn	8.2 mg kg^−1^
Na	180 mg kg^−1^
K	6880 mg kg^−1^
Mg	868 mg kg^−1^
pH	5.76
Aw	0.66

**Table 2:** GA_3_ production in different bioreactors and fermentation techniques using CP or its extract as substrate.

Fermentation	Bioreactor	Time (h)	GA_3_ (mg L^−1^)	Productivity of GA_3_ (mg L^−1^ h^−1^)	GA_3_ (g kg^−1^ of dry CP)	Productivity of GA_3_ (g kg^−1^ h^−1^)
SmF	Erlenmeyer flask	216	236	1.09	2.74	0.01
SmF	STR	96	273	2.84	3.17	0.03
SmF	BCR	216	203	0.94	2.36	0.01
SSSF	Erlenmeyer flask	240	331	1.38	7.69	0.03
SSSF	BCR	216	208	0.96	4.82	0.02
SSF^*∗*^	Erlenmeyer flask	144	946	6.57	7.60	0.05
SSF^*∗*^	Column	120	837	6.97	7.34	0.06

^*∗*^Results of GA_3_ production (mg L^−1^) and productivity (mg L^−1^ h^−1^).
